# Price differences between capsule, menthol non-capsule and unflavoured cigarettes in 65 countries in 2018

**DOI:** 10.1016/j.pmedr.2023.102252

**Published:** 2023-05-19

**Authors:** Nikita B. Rajani, Dickson Qi, Kiara Chang, Christina N. Kyriakos, Filippos T. Filippidis

**Affiliations:** Department of Primary Care and Public Health, School of Public Health, Imperial College London, United Kingdom

**Keywords:** Unflavoured cigarettes, Capsule cigarettes, Menthol cigarettes, Price differentials, Tobacco control

## Abstract

The global consumption of flavoured cigarettes, particularly capsule and menthol non-capsule cigarettes, has been rising rapidly. Their attractiveness has been fuelled by perceptions of improved palatability, along with industry marketing tactics such as lower price points in some regions. This study aimed to compare prices of unflavoured, capsule, and menthol non-capsule cigarettes across 65 countries by analysing 2018 cigarette price data from Euromonitor Passport. Median prices of capsule and menthol non-capsule cigarettes were each compared to unflavoured cigarettes at the country-level. Countries were included in the analysis if they contained price data for capsule or menthol non-capsule and unflavoured cigarettes (n = 65). The median price of capsule cigarettes was the same as unflavoured cigarettes in 12 out of 50 countries and not statistically different in another 31 countries (p > 0.05). Capsule cigarettes were more expensive than unflavoured cigarettes in five countries and cheaper in two (p < 0.05). The median price of menthol non-capsule cigarettes was the same as unflavoured cigarettes in 6 out of 51 countries and not statistically different in another 39 countries (p > 0.05). Menthol non-capsule cigarettes were more expensive than unflavoured cigarettes in five countries and cheaper in one country (p < 0.05). There was no pattern found in the pricing of capsule or menthol non-capsule cigarettes, suggesting variability in the tobacco industry’s pricing strategies across countries. Tailoring tobacco control policies to match national market conditions, particularly in countries with significant market shares of capsule and menthol non-capsule cigarettes could help address the public health threat posed by the tobacco epidemic.

## 1 Introduction

Despite significant progress in tackling the tobacco epidemic, tobacco use continues to pose a significant threat to public health (Kyriakos et al., 2022). The increasing global market shares of tobacco products with added flavour, particularly the most common flavour menthol, is a contributing factor (Kyriakos et al., 2022, Zatoński et al., 2022, Emond et al., 2018). Flavours, such as menthol, can either be added when tobacco is blended, or in the form of a capsule in the filter (Zatoński et al., 2022). In some markets, capsule cigarettes only come in menthol flavour but in other markets a large variety of flavours are available (Kyriakos et al., 2022). The presence of flavour has been found to improve the palatability of cigarettes and has consequently made flavoured cigarettes more appealing among non-smokers and non-regular smokers (Agaku et al., 2015, Barrientos-Gutierrez et al., 2021, Agaku et al.,, Dewhirst and Lee, 2020, Bold et al., 2020). Studies have also found that youths and women tend to perceive cigarettes with added flavour more positively than unflavoured cigarettes, and that the prevalence of such cigarettes is highest in these populations (Agaku et al.,, Dewhirst and Lee, 2020, Bold et al., 2020).

Price is an important determinant of cigarette consumption and often exploited by the tobacco industry to promote cigarette smoking across different demographics. Price promotions which make cigarettes more affordable, especially for flavoured cigarettes, have been strategically applied by the industry to attract price-sensitive smokers such as youths (Emond et al., 2018, Abad-Vivero et al., 2016, Hoek et al., 2019, Gutiérrez-Torres et al., 2021, Sawdey et al., 20202020). For example, in Mexico, after aggressive marketing of lower priced Pall Mall cigarettes, which are primarily flavoured capsule varieties, Pall Mall’s market share of cigarettes increased from 1% to 14% between 2009 and 2016 (Abad-Vivero et al., 2016, Gutiérrez-Torres et al., 2021). Studies in New Zealand and the United States (US) have also revealed that a major reason for young smokers preferring flavoured capsule cigarettes was lower prices compared to unflavoured cigarettes (Emond et al., 2018, Hoek et al., 2019). However, flavoured brands are not consistently cheaper than unflavoured (i.e., standard) brands in all countries. In Chile, the country with the largest domestic market share of flavoured cigarettes, the average unit price for flavoured capsule cigarettes was reportedly 14% higher than that of unflavoured cigarettes (Paraje et al., 2019). In many other countries, particularly across Latin America, rising prices of flavoured capsule cigarettes have been observed despite increasing popularity among price-sensitive youths (Kyriakos et al., 2022, Paraje et al., 2019). Generally, the existing literature on relative prices of capsule cigarettes compared to unflavoured cigarettes is not consistent and often focuses on a singular country or region. The aim of this study was to investigate and compare the prices of capsule, menthol non-capsule and unflavoured cigarettes across a range of countries in different regions of the world. A better understanding of price differences in various countries could help inform public health policies to reduce smoking, particularly among vulnerable populations.

## 2 Methods

We obtained and analysed data from Euromonitor Passport a database from the private market research company, Euromonitor International. Data on Euromonitor Passport is based on various methods such as desk research using official sources (national and industrial), store checks (on place, product, price, and promotion), trade surveys, company analyses, data validation and market analysis (Euro, xxxx). Data from Euromonitor Passport has been frequently used and analysed for tobacco-related research purposes (López-Nicolás and Stoklosa, 2019, Kyriakos et al., 2021).

We extracted cigarette price data for 2018 from all available countries and this included cigarette brand names, pack size, pack price and the unit (per stick) price in both the local currency of the country and US Dollars (USD). We categorised cigarette brands based on specific descriptors in their names (Dewhirst and Lee, 2020, Abad-Vivero et al., 2016, World Health Organisation, 2019, World Health Organisation, 2019). All brands with the descriptors “blast, burst, boost, capsule, caps, cápsula, click, click & roll, crush, crushball, activate, convertibles, choice, change, duo, dual, double, fusion, exchange, hybrid, pop, switch, iSwitch” were categorised as capsule cigarettes. Capsule cigarettes contain one or more capsules in the filter which release flavouring when crushed by the consumer. All brands with the descriptors “green, menthol, menthe, mentol, menta, mentolado, mint” were categorised as menthol non-capsule cigarettes. These cigarettes have an added flavour of menthol which is often associated with reduced harshness and increased palatability (Agaku et al., 2015, Barrientos-Gutierrez et al., 2021, Agaku et al.,, Dewhirst and Lee, 2020, Bold et al., 2020). Brands with a global presence and generally not marketed as flavoured were categorised as unflavoured cigarettes. For example, the brands Benson & Hedges, Camel, Chesterfield, Davidoff, Dunhill, Kent, L&M, Lucky Strike, LD, Marlboro, Pall Mall, Philip Morris, Rothmans, West, and Winston, and brands with no descriptors and brands with the descriptor “red, silver, classic, filter, original, gold”.

Based on the cigarette price per stick provided in local currency, we calculated for each country the median price for each cigarette type (unflavoured, capsule, and menthol non-capsule). Furthermore, we computed for each country the difference in median price of menthol non-capsule cigarettes and capsule cigarettes with respect to unflavoured cigarettes and expressed as a percentage of the median price of unflavoured cigarettes (reference group). Percentages were computed for countries where price data for unflavoured cigarettes and capsule or menthol non-capsule cigarettes was available. The number of observations, median price per unit and range for each category and country are presented in supplementary table 1. Additionally, equality of medians tests for each country were run to test the statistical significance (at the 5% level (0.05)) of any price differences between capsule and unflavoured cigarettes as well as between menthol non-capsule and unflavoured cigarettes (supplementary table 2).

## 3 Results

We identified 4480 price observations from 122 countries, of which 227 observations from 50 countries were capsule brands, ranging from 1 brand in China to 26 in Brazil; 258 observations from 51 countries were menthol non-capsule brands, ranging from 1 brand in Azerbaijan to 30 in Thailand; 598 observations from 65 countries were identified as unflavoured brands, ranging from 2 brands in Azerbaijan to 45 in Australia.

**Fig. 1:**
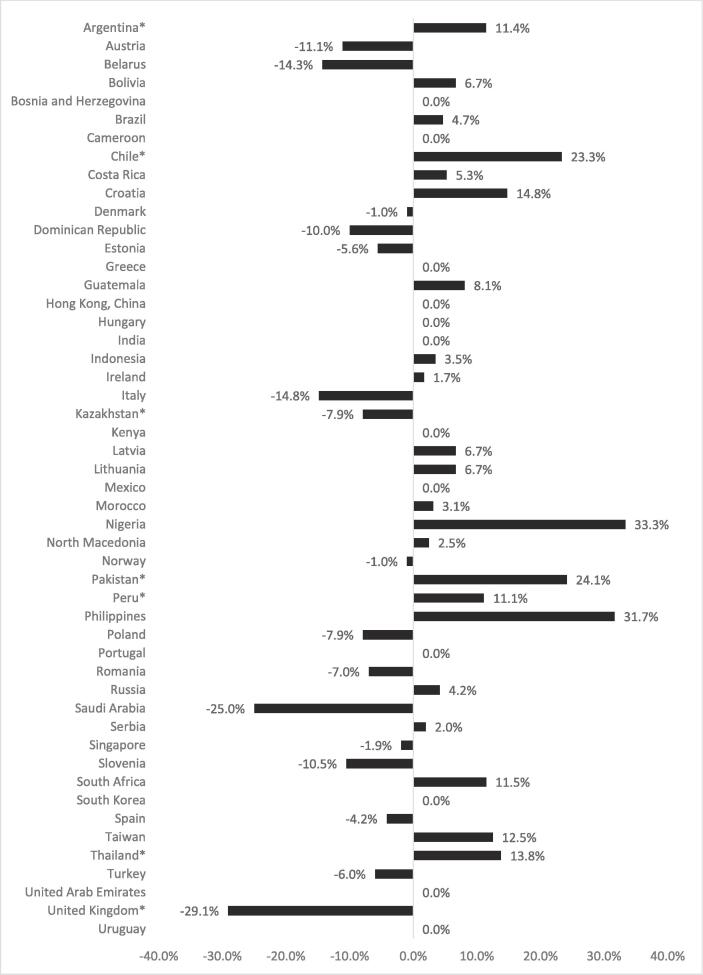
The percentage (%) difference in median unit price between capsule cigarettes and unflavoured cigarettes (reference group) in 50 countries in 2018 *Countries where p-value for equality of medians test was below 0.05.

Fig. 1 presents the percentage difference in the median price of capsule cigarettes compared to unflavoured cigarettes (reference group) in 50 countries in 2018. Among them, 12 countries had the same median price for capsule and unflavoured cigarettes, and an additional 31 countries had no statistically significant difference in median price (p > 0.05). In Argentina, Chile, Pakistan, Peru and Thailand, capsule cigarettes were 11.4%, 23.3%, 24.1%,11.1% and 13.8% more expensive than unflavoured cigarettes, respectively (p < 0.05). Alternatively, the median unit price for capsule cigarettes was 29.1% and 7.9% cheaper in the United Kingdom and Kazakhstan, respectively, than unflavoured cigarettes (p < 0.05).

**Fig. 2:**
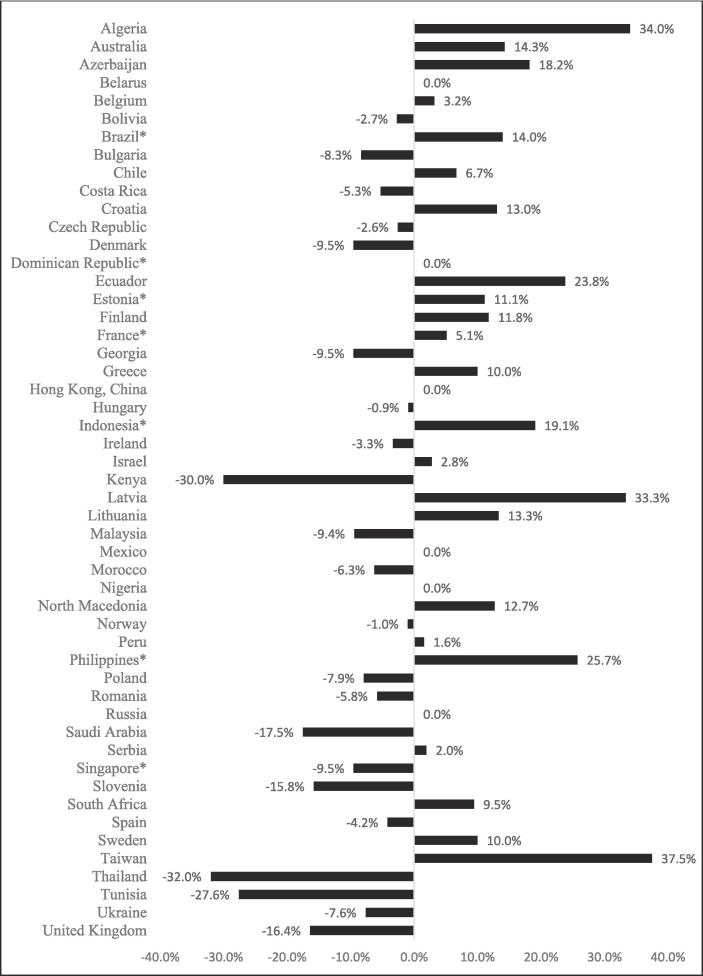
The percentage (%) difference in median unit price between menthol non-capsule cigarettes and unflavoured cigarettes (reference group) in 51 countries in 2018. *Countries where p-value for equality of medians test was below 0.05.

Fig. 2 displays the percentage difference of median unit price between menthol non-capsule and unflavoured cigarettes (reference group) in 2018. Of the 51 countries for which price data was available for both categories, six countries had the same median unit price and an additional 39 countries had no statistically significant difference in median price (p > 0.05). Philippines (25.7%) and Indonesia (19.1%) were the countries where menthol non-capsule cigarettes were the most expensive in comparison to unflavoured cigarettes (p < 0.05). Singapore was the only country where median prices of menthol non-capsule cigarettes was statistically significantly lower than that of unflavoured cigarettes (p < 0.05).

## 4 Discussion

We conducted a cross-sectional analysis comparing the median unit price of both capsule and menthol non-capsule cigarettes with unflavoured cigarettes from 65 different countries. In the majority of countries, the median unit price of capsule and menthol non-capsule cigarettes was similar or lower than of unflavoured cigarettes. However, we found no evidence that capsule and menthol non-capsule cigarettes were consistently cheaper or more expensive than unflavoured cigarettes across the countries in our sample. Price differences between capsule and unflavoured, and menthol non-capsule and unflavoured cigarettes were only statistically significant for a small number of countries. This finding agrees with previous research which showed that in some countries, (e.g., United States), there was little difference between flavoured/menthol and unflavoured cigarettes whilst in other countries (e.g., Chile), flavoured cigarettes were significantly more expensive than standard cigarettes (Agaku et al.,, Gutiérrez-Torres et al., 2021, Sawdey et al., 20202020, Paraje et al., 2019). This seems to suggest that tobacco companies adopt differentiated pricing strategies of flavoured cigarettes, particularly capsule and menthol non-capsule cigarettes, to develop and optimally capture the market (Agaku et al.,, Dewhirst and Lee, 2020, Hoek et al., 2019, Gutiérrez-Torres et al., 2021).

In countries where flavoured cigarettes were cheaper than standard cigarettes, price might be used as a main promotion strategy, intended for price-sensitive individuals such as younger populations. Young smokers in New Zealand and the US were reportedly attracted by the lower prices of flavour capsule cigarettes, but they were unwilling to pay more for this product (Zatoński et al., 2022, Hoek et al., 2019). In Mexico, a low-price strategy employed by Pall Mall substantially boosted its sales (particularly among youths) and market share in the period from 2009 to 2016 (Brown et al., 2021). Similarly, the market share of flavour capsule cigarettes also grew from zero to 18.74% in the same period. Lower price promotions targeting price-sensitive youths can further facilitate smoking initiation and expose young populations to the harmful effects of smoking. Tobacco control policies aiming to protect such susceptible populations could consider restricting discounting strategies and price promotions, in particular for capsule and menthol non-capsule cigarettes, to deter the number of new smokers and reduce the level of tobacco use among young populations (Agaku et al.,).

On the other hand, capsule and menthol non-capsule cigarettes being more expensive than unflavoured cigarettes in some countries could reflect the adoption of an upselling strategy to maximise value captured from existing or regular smokers. In Chile, where the market share of capsule cigarettes ranked the highest globally for the past few years (Paraje et al., 2019), our analysis found that capsule cigarettes were more expensive than unflavoured cigarettes. Paraje et al. (2019) speculated that flavour capsule cigarettes could be targeted at populations with higher incomes (Paraje et al., 2019), whereas other studies attributed flavoured cigarette smoking in Chile to its flavoured cigarette retail environment, particularly unregulated cigarette packaging and point of sale displays (Emond et al., 2018).

In our analysis we have not observed any clear patterns of price differentials between high-, middle- and low-income countries; future studies could explore whether country income level is associated with capsule or menthol non-capsule cigarette consumption. This is the first study to explore price differences of capsule and menthol non-capsule cigarettes and unflavoured cigarettes across many countries. However, there are limitations that should be acknowledged. Whilst there is research exploring more granular pricing variations, such as pricing strategies adopted at sub-national levels, our study did not explore this (Guindon et al., 2020). Furthermore, the dataset used may not have captured all products in each category and consumption levels were not available, which could mean that the calculated median prices might not fully represent the respective national markets. The inclusion of some menthol or flavoured capsule variants in the portfolio of some large brands (e.g., Pall Mall) was not accounted for and should be acknowledged. Similarly, for some countries and categories, the number of observations was small, which may not have allowed for detection of statistically significant price differences. Some categories such as non-capsule cigarettes of other flavours (not menthol) were not part of the analyses due to dataset limitations. Moreover, we did not consider the role of taxes in each country; future research could consider further investigating taxation policies along with prices to provide a clearer picture.

The tobacco industry is adaptive and tailors its approach depending on the context of the respective national tobacco market; tobacco control policies need to account for and align with specific market and price variations as well. Flavoured cigarettes, including flavour capsules and menthol non-capsule cigarettes, have been banned in an increasing number of countries, and evidence supports public health benefits of such policies such as increased quitting (Kyriakos et al.,, Fong et al.,). Although not all countries are considering such bans, our analysis can inform the development of policies which would differentially tax flavoured and unflavoured cigarettes depending on current retail prices and market shares, aiming to maximise the impact of high taxes on cigarette consumption.

## 5 Conclusion

Our analysis found that in the majority of countries there was no significant price difference between capsule and menthol non-capsule cigarettes with unflavoured cigarettes. The tobacco industry tailors the prices of capsule and menthol non-capsule cigarettes to national tobacco markets, where prices are used as either promotion strategies to target price-sensitive populations or as upselling strategies to maximize value from regular smokers. Our findings could be useful to tobacco control policymakers who may wish to target certain types of cigarettes with taxation policies.

## CRediT authorship contribution statement

**Nikita B. Rajani:** Writing – original draft, Writing – review & editing. **Dickson Qi:** Investigation, Methodology, Formal analysis, Writing – original draft. **Kiara Chang:** Conceptualization, Methodology, Writing – review & editing. **Christina N. Kyriakos:** Conceptualization, Methodology, Writing – review & editing. **Filippos T. Filippidis:** Conceptualization, Methodology, Writing – review & editing, Supervision.

## Declaration of Competing Interest

The authors declare that they have no known competing financial interests or personal relationships that could have appeared to influence the work reported in this paper.

## Data Availability

The authors do not have permission to share data.
