# Supplementary material for: Price differences between capsule, menthol non-capsule and unflavoured cigarettes in 65 countries in 2018
Source: Prev Med Rep. 2023 May 19;34:102252. doi: 10.1016/j.pmedr.2023.102252 (PMC10220479; doi:10.1016/j.pmedr.2023.102252)
Supplement: Supplementary data 1 [file mmc1.docx]

Supplementary Table 1. Overview of median unit price (range) in USD for unflavoured, capsule and menthol non-capsule cigarettes

| **Country** | **Unflavoured Cigarettes** | | **Capsule**  **Cigarettes** | | **Menthol Non-Capsule Cigarettes** | |
| --- | --- | --- | --- | --- | --- | --- |
|  | **Obs.**  **(n)** | **Median Unit Price in USD (Range)** | **Obs.**  **(n)** | **Median Unit Price in USD (Range)** | **Obs.**  **(n)** | **Median Unit Price in USD (Range)** |
| Algeria | 8 | 0.11  (0.08 – 0.12) | - | - | 2 | 0.15  (0.06 – 0.23) |
| Argentina | 7 | 0.17  (0.14 – 0.19) | 2 | 0.19  (0.19 – 0.19) | - | - |
| Australia | 45 | 0.80  (0.75 – 1.12) | - | - | 6 | 0.92  (0.80 – 1.30) |
| Austria | 5 | 0.30  (0.27 – 0.32) | 3 | 0.27  (0.27 – 0.30) | - | - |
| Azerbaijan | 2 | 0.06  (0.07 – 0.07) | - | - | 1 | 0.07  (0.07 – 0.07) |
| Belarus | 3 | 0.07  (0.06 – 0.08) | 4 | 0.06  (0.02 – 0.08) | 3 | 0.07  (0.06 – 0.08) |
| Belgium | 9 | 0.36  (0.32 – 0.39) | - | - | 1 | 0.37  (0.37 – 0.37) |
| Bolivia | 3 | 0.11  (0.07 – 0.11) | 3 | 0.12  (0.01 – 0.14) | 3 | 0.11  (0.08 – 0.12) |
| Bosnia & Herzegovina | 8 | 0.15  (0.12 – 0.18) | 1 | 0.15  (0.15 – 0.15) | - | - |
| Brazil | 18 | 0.13  (0.09 – 0.15) | 26 | 0.14  (0.13 – 0.16) | 13 | 0.15  (0.10 – 0.18) |
| Bulgaria | 6 | 0.14  (0.12 – 0.15) | - | - | 1 | 0.13  (0.13 – 0.13) |
| Cameroon | 2 | 0.09  (0.19 – 0.19) | 2 | 0.09  (0.09 – 0.09) | - | - |
| Chile | 12 | 0.23  (0.21 – 0.28) | 15 | 0.29  (0.22 – 0.32) | 2 | 0.25  (0.23 – 0.26) |
| Costa Rica | 1 | 0.17  (0.17 – 0.17) | 1 | 0.18  (0.18 – 0.18) | 1 | 0.16  (0.16 – 0.16) |
| Croatia | 16 | 0.17  (0.12 – 0.21) | 4 | 0.20  (0.17 – 0.21) | 1 | 0.20  (0.20 – 0.20) |
| Czech Republic | 8 | 0.20  (0.19 – 0.21) | - | - | 1 | 0.19  (0.19 – 0.19) |
| Denmark | 11 | 0.32  (0.27 – 0.35) | 13 | 0.31  (0.28 – 0.35) | 9 | 0.29  (0.27 – 0.35) |
| Dominican Republic | 7 | 0.21  (0.21 – 0.42) | 17 | 0.19  (0.19 – 0.42) | 11 | 0.21  (0.02 – 0.42) |
| Ecuador | 7 | 0.21  (0.13 – 0.34) | - | - | 1 | 0.26  (0.26 – 0.26) |
| Estonia | 4 | 0.20  (0.19 – 0.21) | 2 | 0.20  (0.20 – 0.20) | 2 | 0.23  (0.23 – 0.23) |
| Finland | 2 | 0.39  (0.37 – 0.40) | - | - | 1 | 0.42  (0.42 – 0.42) |
| France | 11 | 0.44  (0.43 – 0.45) | - | - | 4 | 0.46  (0.44 – 0.47) |
| Georgia | 9 | 0.08  (0.07 – 0.16) | - | - | 3 | 0.08  (0.08 – 0.16) |
| Greece | 9 | 0.23  (0.22 – 0.25) | 3 | 0.23  (0.23 – 0.24) | 1 | 0.25  (0.25 – 0.25) |
| Guatemala | 8 | 0.14  (0.12 – 0.16) | 1 | 0.15  (0.15 – 0.15) | - | - |
| Hong Kong, China | 9 | 0.37  (0.35 – 0.40) | 2 | 0.37  (0.37 – 0.37) | 9 | 0.37  (0.32 – 0.38) |
| Hungary | 14 | 0.21 (0.19 – 0.24) | 6 | 0.21  (0.19 – 0.22) | 14 | 0.21  (0.19 – 0.23) |
| India | 5 | 0.23 (0.19 – 0.23) | 3 | 0.23  (0.23 – 0.23) | - | - |
| Indonesia | 3 | 0.08  (0.07 – 0.09) | 3 | 0.08  (0.08 – 0.10) | 2 | 0.10  (0.10 – 0.10) |
| Ireland | 9 | 0.68  (0.58 – 0.73) | 3 | 0.69  (0.68 – 0.73) | 8 | 0.67  (0.59 – 1.07) |
| Israel | 16 | 0.40  (0.28 – 0.49) | - | - | 5 | 0.41  (0.39 – 0.45) |
| Italy | 4 | 0.30  (0.28 – 0.32) | 3 | 0.26  (0.26 – 0.28) | - | - |
| Kazakhstan | 2 | 0.06  (0.06 – 0.06) | 2 | 0.06  (0.06 – 0.06) | - | - |
| Kenya | 3 | 0.12  (0.10 – 0.14) | 1 | 0.12  (0.12 – 0.12) | 2 | 0.09  (0.05 – 0.12) |
| Latvia | 13 | 0.17  (0.15 – 0.21) | 2 | 0.18  (0.17 – 0.19) | 2 | 0.22  (0.21 – 0.23) |
| Lithuania | 13 | 0.17  (0.15 – 0.20) | 2 | 0.19  (0.17 – 0.20) | 7 | 0.19  (0.17 – 0.21) |
| Malaysia | 9 | 0.20  (0.14 – 0.20) | - | - | 12 | 0.18  (0.13 – 0.20) |
| Mexico | 15 | 0.14  (0.12 – 0.14) | 5 | 0.20  (0.05 – 0.14) | 4 | 0.14  (0.10 – 0.14) |
| Morocco | 11 | 0.16  (0.10 – 0.18) | 1 | 0.17  (0.17 – 0.17) | 3 | 0.15  (0.15 – 0.18) |
| Nigeria | 8 | 0.04  (0.02 – 0.05) | - | - | 4 | 0.04  (0.03 – 0.05) |
| North Macedonia | 16 | 0.09  (0.06 – 0.13) | 1 | 0.09  (0.09 – 0.09) | 1 | 0.10  (0.10 – 0.10) |
| Norway | 12 | 0.75  (0.71 – 0.76) | 6 | 0.74  (0.73 – 0.76) | 4 | 0.74  (0.74 – 0.75) |
| Pakistan | 22 | 0.07  (0.02 – 0.07) | 8 | 0.09  (0.08 – 0.09) | - | - |
| Peru | 7 | 0.19  (0.12 – 0.21) | 3 | 0.21  (0.21 – 0.22) | 2 | 0.20  (0.19 – 0.20) |
| Philippines | 22 | 0.07  (0.04 – 0.11) | 1 | 0.09  (0.09 – 0.09) | 11 | 0.08  (0.06 – 0.11) |
| Poland | 13 | 0.20  (0.17 – 0.22) | 1 | 0.19  (0.19 – 0.19) | 5 | 0.19  (0.17 – 0.22) |
| Portugal | 5 | 0.25  (0.24 – 0.26) | 2 | 0.25  (0.24 – 0.25) | - | - |
| Romania | 4 | 0.22  (0.20 – 0.25) | 5 | 0.20  (0.19 – 0.21) | 4 | 0.20  (0.20 – 0.22) |
| Russia | 15 | 0.10  (0.08 – 0.12) | 8 | 0.11  (0.08 – 0.12) | 8 | 0.10  (0.09 – 0.12) |
| Saudi Arabia | 13 | 0.32  (0.19 – 0.40) | - | - | 2 | 0.27  (0.20 – 0.33) |
| Serbia | 14 | 0.12  (0.11 – 0.15) | 1 | 0.12  (0.12 – 0.12) | 3 | 0.12  (0.12 – 0.13) |
| Singapore | 16 | 9.52  (8.40 – 10.28) | 10 | 9.34  (8.40 – 10.79) | 14 | 8.62  (8.40 – 10.28) |
| Slovenia | 14 | 0.21  (0.19 – 0.23) | 1 | 0.20  (0.20 – 0.20) | 3 | 0.19  (0.19 – 0.20) |
| South Africa | 9 | 0.11  (0.09 – 0.17) | 4 | 0.12  (0.11 – 0.13) | 8 | 0.13  (0.10 – 0.15) |
| South Korea | 4 | 0.20  (0.20 – 0.20) | 2 | 0.20  (0.20 – 0.20) | - | - |
| Spain | 5 | 0.27  (0.24 – 0.29) | 2 | 0.26  (0.26 – 0.26) | 1 | 0.26  (0.26 – 0.26) |
| Sweden | 3 | 0.33  (0.31 – 0.37) | - | - | 2 | 0.36  (0.34 – 0.37) |
| Taiwan | 3 | 0.13  (0.13 – 0.16) | 7 | 0.15  (0.15 – 0.21) | 10 | 0.18  (0.11 – 0.23) |
| Thailand | 9 | 0.21  (0.09 – 0.22) | 1 | 0.24  (0.24 – 0.24) | 30 | 0.15  (0.09 – 0.22) |
| Tunisia | 4 | 0.12  (0.11 – 0.13) | - | - | 2 | 0.08  (0.06 – 0.10) |
| Turkey | 5 | 0.14  (0.12 – 0.15) | 3 | 0.13  (0.13 – 0.14) | - | - |
| United Arab Emirates | 2 | 0.27  (0.27 – 0.27) | 1 | 0.27  (0.27 – 0.27) | - | - |
| Ukraine | 3 | 0.06  (0.06 – 0.06) | - | - | 1 | 0.06  (0.06 – 0.06) |
| United Kingdom | 18 | 0.71  (0.48 – 0.74) | 17 | 0.50  (0.47 – 0.64) | 8 | 0.60  (0.49 – 0.74) |
| Uruguay | 5 | 0.24  (0.23 – 0.26) | 1 | 0.24  (0.24 – 0.24) | - | - |
| **All countries** | **598** | **0.20**  **(0.02 – 10.28)** | **227** | **0.20**  **(0.02 – 10.79)** | **258** | **0.19**  **(0.02 – 10.28)** |

Supplementary Table 2. Median unit price difference (%) between capsule cigarettes or menthol non-capsule cigarettes and unflavoured cigarettes in 65 countries

| **Country** | **Capsule Cigarettes vs. Unflavoured Cigarettes**  **(n=50)** | | **Menthol Non-Capsule Cigarettes vs. Unflavoured Cigarettes (n=51)** | |
| --- | --- | --- | --- | --- |
|  | **Median Unit Price Difference (%)** | **Equality of Medians Test P-Value** | **Median Unit Price Difference (%)** | **Equality of Medians Test P-Value** |
| Algeria | - | - | 34.0 | >0.999 |
| Argentina | 11.4 | 0.023 | - | - |
| Australia | - | - | 14.3 | 0.058 |
| Austria | -11.1 | 0.408 | - | - |
| Azerbaijan | - | - | 18.2 | >0.999 |
| Belarus | -14.3 | 0.809 | 0.0 | >0.999 |
| Belgium | - | - | 3.2 | 0.292 |
| Bolivia | 6.7 | 0.083 | -2.7 | 0.414 |
| Bosnia & Herzegovina | 0.0 | 0.343 | - | - |
| Brazil | 4.7 | 0.548 | 14.0 | <0.001 |
| Bulgaria | - | - | -8.3 | 0.350 |
| Cameroon | 0.0 | >0.999 | - | - |
| Chile | 23.3 | 0.001 | 6.7 | 0.825 |
| Costa Rica | 5.3 | 0.157 | -5.3 | 0.157 |
| Croatia | 14.8 | 0.264 | 13.0 | 0.274 |
| Czech Republic | - | - | -2.6 | 0.343 |
| Denmark | -1.0 | 0.682 | -9.5 | 0.653 |
| Dominican Republic | -10.0 | 0.088 | 0.0 | 0.093 |
| Ecuador | - | - | 23.8 | 0.285 |
| Estonia | -5.6 | 0.221 | 11.1 | 0.014 |
| Finland | - | - | 11.8 | 0.083 |
| France | - | - | 5.1 | 0.012 |
| Georgia | - | - | -9.5 | 0.735 |
| Greece | 0.0 | 0.735 | 10.0 | 0.292 |
| Guatemala | 8.1 | 0.134 | - | - |
| Hong Kong, China | 0.0 | 0.338 | 0.0 | 0.257 |
| Hungary | 0.0 | >0.999 | -0.9 | >0.999 |
| India | 0.0 | >0.999 | - | - |
| Indonesia | 3.5 | 0.414 | 19.1 | 0.025 |
| Ireland | 1.7 | 0.505 | -3.3 | 0.772 |
| Israel | - | - | 2.8 | 0.882 |
| Italy | -14.8 | 0.147 | - | - |
| Kazakhstan | -7.9 | 0.046 | - | - |
| Kenya | 0.0 | 0.505 | -30.0 | 0.361 |
| Latvia | 6.7 | 0.756 | 33.3 | 0.104 |
| Lithuania | 6.7 | 0.756 | 13.3 | 0.423 |
| Malaysia | - | - | -9.4 | 0.528 |
| Mexico | 0.0 | >0.999 | 0.0 | >0.999 |
| Morocco | 3.1 | 0.217 | -6.3 | 0.923 |
| Nigeria | 33.3 | 0.119 | 0.0 | >0.999 |
| North Macedonia | 2.5 | 0.506 | 12.7 | 0.163 |
| Norway | -1.0 | 0.457 | -1.0 | 0.182 |
| Pakistan | 24.1 | 0.001 | - | - |
| Peru | 11.1 | 0.011 | 1.6 | 0.858 |
| Philippines | 31.7 | 0.286 | 25.7 | 0.003 |
| Poland | -7.9 | 0.369 | -7.9 | 0.308 |
| Portugal | 0.0 | 0.290 | - | - |
| Romania | -7.0 | 0.099 | -5.8 | 0.157 |
| Russia | 4.2 | 0.907 | 0.0 | 0.472 |
| Saudi Arabia | -25.0 | 0.710 | -17.5 | 0.591 |
| Serbia | 2.0 | 0.464 | 2.0 | 0.938 |
| Singapore | -1.9 | >0.999 | -9.5 | 0.024 |
| Slovenia | -10.5 | 0.464 | -15.8 | 0.218 |
| South Africa | 11.5 | 0.165 | 9.5 | 0.819 |
| South Korea | 0.0 | >0.999 | - | - |
| Spain | -4.2 | 0.290 | -4.2 | 0.439 |
| Sweden | - | - | 10.0 | 0.709 |
| Taiwan | 12.5 | 0.778 | 37.5 | 0.067 |
| Thailand | 13.8 | 0.035 | -32.0 | 0.409 |
| Tunisia | - | - | -27.6 | 0.083 |
| Turkey | -6.0 | 0.465 | - | - |
| United Arab Emirates | 0.0 | >0.999 | - | - |
| Ukraine | - | - | -7.6 | 0.247 |
| United Kingdom | -29.1 | <0.001 | -16.4 | 0.919 |
| Uruguay | 0.0 | 0.624 | - | - |
